# Nanoparticle-Based Biosensing of Tuberculosis, an Affordable and Practical Alternative to Current Methods

**DOI:** 10.3390/bios9010001

**Published:** 2018-12-24

**Authors:** Nirajan Bhusal, Sunaina Shrestha, Nisha Pote, Evangelyn C. Alocilja

**Affiliations:** 1 Dhulikhel Hospital, Kathmandu University, Dhulikhel 45200, Kavrepalanchok, Nepal; nirajanbhusal@gmail.com (N.B.); stha.sunaina@gmail.com (S.S.); potenisha@gmail.com (N.P.); 2 Global Alliance for Rapid Diagnostics, Michigan State University, East Lansing, MI 48824, USA; 3 Nano-Biosensors Lab, Michigan State University, East Lansing, MI 48824, USA

**Keywords:** Colorimetric assay, sputum microscopy, Expert MTB/RIF, acid-fast bacilli, pulmonary TB

## Abstract

Access to community-based point-of-care, low-cost, and sensitive tuberculosis (TB) diagnostics remains an unmet need. Objective: The objective of this study was to combine principles in nanotechnology, TB biology, glycochemistry, and engineering, for the development of a nanoparticle-based colorimetric biosensing assay (NCBA) to quickly and inexpensively detect acid-fast bacilli (AFB) in sputum samples. Methods: In NCBA, the isolation of AFB from sputum samples was accomplished through glycan-coated magnetic nanoparticles (GMNP) interacting with AFB and then using a simple magnet to separate the GMNP-AFB complex. Acid-fastness and cording properties of mycobacteria were utilized to provide visually observable red-stained clumps of bacteria that were surrounded by brown nanoparticles under a light microscope on prepared smears. The NCBA technique was compared against sputum smear microscopy (SSM) and Xpert MTB/RIF in 500 samples from patients that were suspected to have TB. Results: Statistical analysis showed that NCBA had sensitivity and specificity performances in perfect agreement with Xpert MTB/RIF as gold standard for all 500 samples. SSM had a sensitivity of 40% for the same samples. Conclusion: NCBA technique yielded full agreement in terms of sensitivity and specificity with the Xpert MTB/RIF in 500 samples. The method is completed in 10–20 min through a simple process at an estimated cost of $0.10 per test. Implementation of NCBA in rural communities would help to increase case finding and case notification, and would support programs against drug-resistance. Its use at the first point-of-contact by patients in the healthcare system would facilitate quick treatment in a single clinical encounter, thus supporting the global “End TB Strategy” by 2035.

## 1. Introduction

Despite decades of effective treatment being available, tuberculosis (TB) is still in the top 10 causes of death worldwide [1]. It is caused by the bacteria *Mycobacterium tuberculosis* (Mtb), which most often affect the lungs. In 2016, 10.4 million people got sick of TB and 1.7 million died from the disease, of which 95% occurred in low- and medium-income countries [1]. About one-fourth of the human population is infected with latent TB and 5–15% of this population has a lifetime risk of falling ill with TB [1].

In Nepal, TB is the sixth leading cause of death in the country [2]. It has been estimated that 44,000 people develop active TB every year and 20,500 have infectious pulmonary disease that can spread to others [2]. In a 2017 national report, TB deaths in Nepal reached 5506, accounting for 3.5% of all deaths and placing Nepal as the 43rd most affected country in the world [3]. The diagnosis and treatment monitoring of TB patients in Nepal relies on sputum smear microscopy, because it is easy to administer and inexpensive. The country has 596 microscopy centers that carry out sputum microscopy examinations, mostly in government health facilities (487) with the remainder in non-profit organizations and private institutions [3].

For decades, many countries have relied on direct (un-concentrated) sputum smear microscopy (SSM) as the primary method for detecting pulmonary TB. SSM is the first microbial analysis both for tuberculosis diagnosis and assessment of patient infectiousness, which is used as a guide to isolation measures and contact investigations [4,5,6,7,8]. The method is fast, simple, inexpensive, and specific for Mtb in areas of high prevalence [9,10]. SSM is widely applicable in various populations with different socio-economic conditions [9]. However, it has significant limitations in its performance. The sensitivity is compromised when bacterial load is less than 10,000 organisms/mL sputum sample [9,10]. SSM also has a poor track record in extra-pulmonary tuberculosis, pediatric tuberculosis, and in TB patients suffering from the common comorbidity of human immunodeficiency virus (HIV) [9]. Due to the requirement of serial sputum examinations, some patients do not come back for the repeated sampling or for the exam results, leading to lost opportunity for treatment and follow up. It has been reported that many of the TB diagnoses happen after multiple health care visits and lengthy delays, with an average delay of 28–30 days from the patient’s first contact of a healthcare provider to diagnosis [11].

Recently, several methods have been developed for the diagnosis and concentration of TB and multi-drug resistant tuberculosis (MDR-TB), such as Xpert MTB/RIF, TB beads, liquid culture, centrifugation, and filtration [12,13,14,15,16]. While these diagnostic methods are more sensitive and/or specific than SSM, they are oftentimes prohibitively expensive and not too accessible for those living in low-resource countries where Mtb has a high prevalence.

The World Health Organization recommended the Xpert MTB/RIF in 2010 to diagnose all persons with signs and symptoms of TB. In 2016, the public sector procured 6.9 million cartridges under concessional pricing, but this is still far less than the number that would be needed to diagnose the more than 10 million people who fall ill with TB each year [11].

The difficulty faced by TB care and prevention efforts has been the issue of rapid, reliable, and universally accessible diagnosis. Rapid TB diagnosis is one of the most important steps for instituting control measures. Faster TB diagnosis means earlier treatment and epidemiological control. Without proper diagnosis, disease treatment can become a trial-and-error effort. Unfortunately, accurate and timely diagnosis of TB is still wanting.

The ambitious goal of the global “End TB Strategy” to reduce TB incidence to 90% and reduce TB mortality to 95% by 2035 will not be achieved without new tools to fight TB [17]. These tools include improved point-of-care diagnostic tests that can be delivered in communities and at the first point-of-contact by patients in the healthcare system [17]. These tests should be done on an easily accessible sample and results be provided in a timely manner, allowing for a quick turnaround time for treatment in a single clinical encounter, hence avoiding loss of patient follow up [17].

One of the most important research developments in modern science is nanotechnology. It allows scientists, engineers, chemists, and physicians to work at the molecular and cellular levels to produce significant advances in the life sciences and healthcare. A new method of detecting TB in pulmonary cases that are based on nanoparticle science is presented in this paper. The objective of this study was to combine principles in nanotechnology, TB biology, glycochemistry, and engineering for the development of a nanoparticle-based colorimetric biosensing assay (NCBA) to quickly and inexpensively detect very low concentrations of acid-fast bacilli (AFB) in sputum samples. The novelty of NCBA includes the utilization of iron oxide nanoparticles with superparamagnetic properties. The use of magnetic nanoparticles (MNPs) offers major advantages due to their unique size and physicochemical properties. The MNP solution is colloidal in nature and it provides stability, which gives rise to both steric and coulombic repulsions. Their nanoscale size results in their higher effective surface areas, lower sedimentation rates, and minimal precipitation due to gravitation forces [18]. The MNPs are coated with glycan to facilitate attachment on the bacterial cell wall through carbohydrate-binding protein sites, providing specificity to the biosensing mechanism. In this study, NCBA’s detection performance was compared with the widely used SSM method and the WHO-recommended Xpert MTB/RIF system.

## 2. Materials and Methods

### 2.1. Chemicals and Reagents

Carbol fuchsin (0.3%, primary stain) was prepared by dissolving 50 g phenol in 100 mL 90% ethanol and then adding 3 g basic fuchsin in the mixture. Distilled water was added to bring the total volume to 1 L. The decolorization solution was 25% sulphuric acid. The counter stain was 0.3% methylene blue. Xpert MTB/RIF reagent from the kit (Cepheid, Sunnyvale, CA, USA) was used to decontaminate and digest the mucoid sputum sample.

Glycan-coated magnetic nanoparticles (GMNPs) were provided by the Alocilja Research Group from Michigan State University (East Lansing, Michigan, USA). GMNPs consisted of iron oxide (III) or magnetite (Fe_3_O_4_) core and a glycan (chitosan) shell. Fe_3_O_4_ was synthesized using ferric chloride hexahydrate (FeCl_3_·6H_2_O) as precursor in a mixture of ethylene glycol (as reducing agent) and sodium acetate (as porogen). Chitosan was polymerized to surface-modify the iron oxide nanoparticles. Glycan coating on MNP allows for the simple and inexpensive capture of Mtb cells through glycan-glycoprotein interaction without the use of expensive antibodies or aptamers. The Mtb cell envelope is rich in complex carbohydrates and glycoproteins that bind with the glycan functionalized nanoparticles. While glycan-glycoprotein interaction is not necessarily specific to pulmonary *M. tuberculosis*, environmental and non-pathogenic mycobacteria do not usually manifest in pulmonary sputum samples.

### 2.2. Instrumentation

Instruments used in the study included Xpert MTB/RIF polymerase chain reaction (PCR) machine (Cepheid, Sunnyvale, CA, USA), bright field microscope (Olympus CX41, Japan Inc.), and a three-dimensional (3-D) printed magnetic rack containing Neodymium magnets.

### 2.3. Clinical Samples

In this study, leftover pulmonary samples that were collected between January 2018 and April 2018 sent to the Department of Medical Microbiology, Kathmandu University Dhulikhel Hospital, were investigated. These samples were spontaneously produced sputum specimens early in the morning from study patients for three successive days. Sputum of patients clinically suspected of TB having symptoms, such as coughing for about two weeks, fever, weight loss of greater than 3 kg or dyspnea, or having radiographic imaging features of TB, were collected in sterile screw-capped containers prior to treatment. A total of 500 sputum specimens from 500 TB-symptomatic patients were used in the study. There were no samples from patients asymptomatic of TB or unrelated to TB.

### 2.4. Sputum Sample Processing

**Figure 1:**
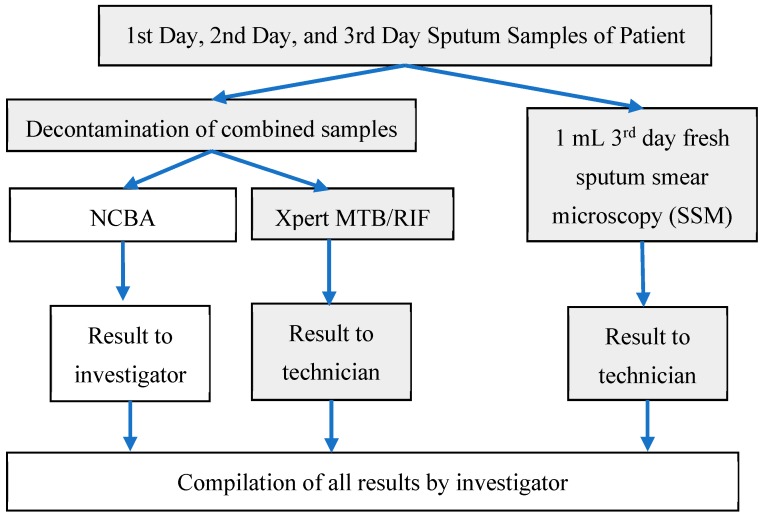
Schematic diagram of sample handling and processing. Activities conducted by the technician are identified as grey boxes while activities conducted by the investigator are white boxes. Sputum sample obtained on the third day from the same patient was tested for AFB by SSM and the remainder was combined with the earlier two samples for testing by NCBA and Xpert MTB/RIF.

Sputum samples were tested for TB using three techniques: conventional sputum smear microscopy (SSM), Xpert MTB/RIF as the gold standard, and the nanoparticle-based colorimetric biosensing assay (NCBA). Sputum samples collected from a patient in the first two days were stored in the refrigerator. Sputum sample obtained on the third day from the same patient was tested for the presence of acid-fast bacilli (AFB) by SSM and the remainder was combined with the earlier two samples amounting to about 4 milliliters (mL). The combined sputum specimen was divided into two portions and subjected to the Xpert MTB/RIF and NCBA technique. A schematic diagram of the sample processing protocol is presented in Figure 1.

Sample testing and data collection for Xpert MTB/RIF and SSM were blinded to the investigator, that is, SSM and Xpert testing were done by a lab technologist while the NCBA testing was conducted by the investigator. The three testing techniques are described, as follows:

1. Sputum Smear Microscopy (SSM): A smear was prepared by the Ziehl–Neelsen (ZN) method of the third (fresh) sputum sample following the standard protocol by the International Union Against Tuberculosis and Lung Disease [19] within 2 h after receipt at the hospital. Briefly, about 20 µL of fresh sputum sample was placed on a glass slide and heat fixed by a passing flame from a Bunsen burner. The slide was placed on a staining rack and 0.3% carbol fuchsin was poured over the smear. The underside of the slide was gently heated by passing a flame under the rack until fumes appeared. After cooling for about 5 min, the smear was rinsed with distilled water until no color appeared in the effluent. The smear was washed with 25% sulphuric acid several times until the smear appeared light pink in color. The smear was washed with distilled water and then the counter stain (0.3% methylene blue) was added to cover the smear. Distilled water was used to wash off the counter stain and then the smear was air-dried. Once ready, the smear was examined under a light microscope using 100× oil immersion objective to observe the presence of red-colored AFB.
2. Xpert MTB/RIF (Cepheid, Sunnyvale, CA, USA): Decontamination reagent from the kit was added to the combined sputum sample in a 2:1 (reagent to sample) ratio in a 15-mL falcon tube, which was then manually agitated twice (or quick vortex) during a 15-min incubation period at room temperature. Subsequently, 2 mL of the decontaminated sputum sample were transferred to the test cartridge using a sterile disposable pipette (provided with the kit). The cartridge was loaded into the Xpert MTB/RIF PCR machine and operated for 1 hour and 50 min. At the end of the real-time PCR run, the result was generated [20].
3. Nanoparticle-based Colorimetric Biosensing Assay (NCBA): About 1 mL of the decontaminated sputum sample was added into a 1.5 mL tube containing 100 µL of the nanoparticles. The GMNP and sputum were mixed and allowed to incubate for 5 min at room temperature. The tube was then placed in a magnetic rack to separate the magnetic GMNP-AFB complex and the supernatant was discarded. A smear was prepared by adding 20 µL of the concentrated GMNP-AFB complex on a glass slide following the SSM procedure, as described in Section 1 above.

### 2.5. Statistical Analysis

Using Xpert MTB/RIF results as the gold standard, sensitivity, specificity, positive predictive value (PPV), negative predictive value (NPV), and accuracy for SSM and NCBA were calculated at 95% confidence interval (CI) using the online MedCalc statistical software (https://www.medcalc.org/calc/diagnostic_test.php).

## 3. Results

**Figure 2:**
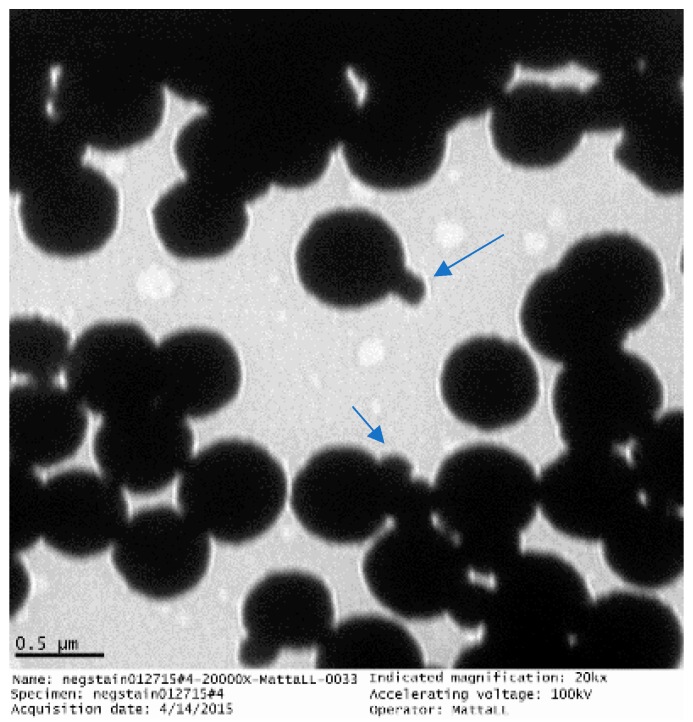
Transmission electron microscope (TEM) image of glycan-coated magnetic nanoparticle clusters, with several iron oxides enclosed in the glycan polymer. Some nanoparticles are protruding from the cluster (See arrows). (TEM image by the Nano-Biosensors Lab, Michigan State University.)

The synthesized GMNPs occur in clusters with several iron oxide nanoparticles being enclosed in the glycan polymer, as shown in Figure 2. The iron oxide nanoparticles have an average size of 99±58 nm. These GMNPs are superparamagnetic, that is, their magnetization on average is zero, but an external field is able to magnetize the nanoparticles. The prepared GMNP for this study has been stored at room temperature and it has been stable for at least 12 months.

**Figure 3:**
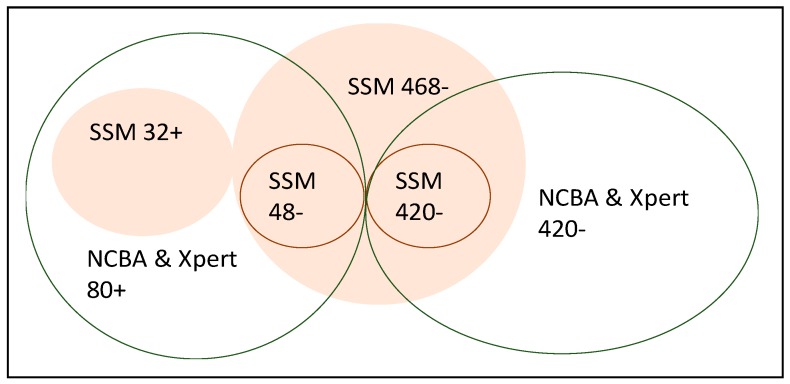
Result of the 500-sample analysis. Sputum Smear Microscopy (SSM) is shaded while nanoparticle-based colorimetric biosensing assay (NCBA) and Xpert MTB/RIF are not shaded. For the SSM test, 32 were positive (32+) and 468 were negative (468−); The 32+ SSM samples were all positive in Xpert and NCBA. Of the 468− (negative) SSM samples, 48 were positive and 420 were negative in both NCBA and Xpert MTB/RIF.

Five hundred sputum samples that were obtained from 500 patients were included in the study. All 500 samples were tested for TB using SSM, Xpert MTB/RIF, and NCBA. For the SSM test, 32 were positive (32+) and 468 were negative (468−); for the Xpert test, 80 were positive (80+) and 420 were negative (420−); and, for the NCBA test, 80 were positive (80+) and 420 were negative (420−). The 32+ SSM samples were all positive in Xpert and NCBA. Of the 468− (negative) SSM samples, 48 were positive in both Xpert and NCBA. Figure 3 shows a Venn diagram of the distribution of the results from the three tests. Table 1 presents the results from SSM test (not shaded) and NCBA test (shaded) using Xpert MTB/RIF as the gold standard in defining the number of true TB cases and non-TB cases.

Statistical analysis of the diagnostic comparison between SSM and NCBA, using Xpert MTB/RIF results as gold standard for true cases, are presented in Table 2. At 95% confidence interval, the results show that SSM has a sensitivity of only 40% (29%∓52%), while NCBA has a sensitivity matching that of the Xpert system (95%∓100%). Probably because sputum samples were from suspected TB patients, the specificity, positive predictive value (PPV), and negative predictive value (NPV), for SSM and NCBA are very high close to 100%. The accuracy of SSM is 90% (87∓93%), while the accuracy of NCBA is 100% (99%∓100%). Given the sample size and nature of the collected samples, the calculated prevalence for this cohort of patients is 16% (80 out of 500).

**Figure 4:**
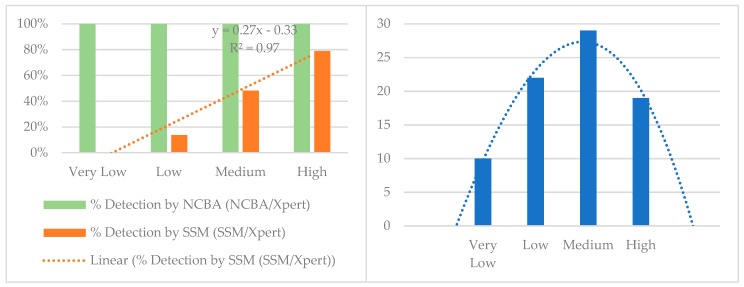
(**Left**) Percent detection by NCBA and SSM with respect to the Xpert MTB/RIF system. SSM detection increases linearly with acid-fast bacillus (AFB) concentration in the sputum sample. (**Right**) TB positive samples are normally distributed around the medium level.

The Xpert MTB/RIF system reports bacterial load as very low, low, medium, and high. These categories were used to estimate the bacterial load in SSM and NCBA by matching the corresponding samples with the Xpert system. Table 3 shows a comparison of the detection limit and dynamic range of detection of the two techniques with respect to the Xpert system. Results from NCBA match well with the results from the Xpert MTB/RIF at all levels. On the other hand, SSM could not detect at very low level, could detect only 14% at low level, 48% at medium level, and 79% at high level. A graphical analysis was conducted on the SSM and NCBA results with respect to Xpert MTB/RIF as the gold standard and the results are presented in Figure 4. Sensitivity of NCBA matches well with the Xpert system at all bacterial loads, while SSM increases linearly with increasing bacterial load (y = 0.27x − 0.33, R^2^ = 0.97, Figure 4 Left). TB positive samples are normally distributed around the medium level (Figure 4 Right). SSM has 48% detection at the medium level, which is very close to the calculated sensitivity of 40% (29–52%) in Table 2.

**Figure 5:**
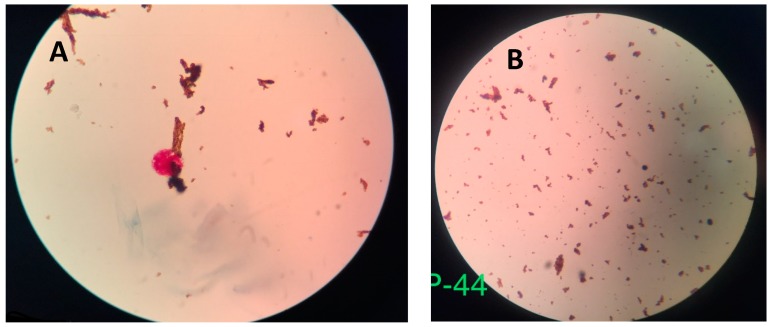
(**A**) TB positive sample: Clumped red glycan-coated magnetic nanoparticles (GMNP)-AFB complex surrounded by brown GMNPs. (**B**) TB negative sample: Dispersed brown GMNP.

Figure 5 shows typical NCBA results for TB+ and TB− sputum samples, as viewed through the eyepiece of the bright field microscope. A TB+ sample (Figure 5A) shows clumped red GMNP-AFB complexes surrounded by dull brown nanoparticles, while a TB- sample (Figure 5B) shows dispersed brown nanoparticles.

## 4. Discussion

### 4.1. GMNP-AFB Complex

The interaction of GMNP and AFB forming the GMNP-AFB complex is part of the novelty in the NCBA technique. It is the foundation for the isolation of AFB from the sputum sample. It is also the foundation for increased sensitivity, as few GMNPs can remove and concentrate a larger number of bacteria due to the cording phenomenon of AFB. Cording is the formation of tight bundles or aggregation of cells in a definite order. GMNPs on cell walls can extract multiple corded bacteria. Once bound, the GMNP-AFB complexes are isolated and concentrated from the rest of the sample matrix by applying a magnetic field through a simple magnet. The GMNP-AFB complexes are acid-fast stained and color is observed. Positive samples show red clumps (corded and agglomerated) that are surrounded by brown GMNPs while negative samples show dispersed spatially distributed dull brown GMNPs.

The GMNP-AFB complex is hypothesized to occur through interaction between glycans and glycan-binding proteins on bacterial cell surface. There are two classes of glycan-binding proteins: lectins and glycosaminoglycan-binding proteins [21]. Lectins tend to recognize specific terminal aspects of glycan chains by fitting them into well-defined but shallow binding pockets, while protein interactions with sulfated glycosaminoglycans seem to involve surface clusters of positively charged amino acids [21]. Binding sites in lectins are largely pre-organized shallow grooves and no major structural rearrangement of the proteins is observed upon complex formation [22]. Lectins have broad specificities for oligosaccharides [22]. Park et al. showed that laboratory-prepared fluorescent magnetic glyconanoparticles were able to identify lectins that were displayed on pathogenic and mammalian cell surfaces [23]. As the research is a proof-of-concept for NCBA, specificity/cross reactivity experiments were not conducted, however, they will become part of future studies.

### 4.2. Novel Colorimetric Biosensing Mechanism

On 24 March 1882, Robert Koch gave his breakthrough lecture on the discovery of the bacillus *M. tuberculosis*, in which he described the first successful attempt to stain *M. tuberculosis* by spreading mycobacteria-infected specimens on coverslips [24]. The staining discovery was followed by several modifications by P. Ehrlich, Ziehl, and Rindfleisch in the same year and by Neelsen in the following year [24], and finally becoming into what we now know as the Ziehl-Neelsen staining method. From then on, *M. tuberculosis* would be known as an acid-fast bacillus (AFB) for its ability to resist decolorization by ethanolic acid wash [24]. Acid-fast bacteria have a high concentration of the lipid mycolic acid in their walls along with other kinds of lipids. Once the stain goes into the wall, the cell does not destain or decolorize easily. Red stain from Ziehl-Neelsen facilitate the colorimetric detection; magnetic nanoparticles extract and concentrate the bacteria; and, cording improves the visibility of AFB on smear. Thus, the acid-fastness and cording properties of AFB, coupled with centrifuge-free extraction and concentration by the glycan-nanoparticles, form the novel biosensing mechanism of NCBA.

### 4.3. SSM vs. Xpert MTB/RIF

Out of the 500 samples, 32 samples were positive in SSM, while 80 samples were positive in Xpert MTB/RIF, generating a sensitivity of 40%. For SSM, Singhal and Myneedu [12] reported sensitivities of 22–43% [12], while Laserson et al. reported a sensitivity of 34% [25]. Forty-eight samples were positive in Xpert MTB/RIF while negative in SSM; thus, all 48 samples were considered to be false negative in SSM. False negative results contribute to increased TB transmission in communities and potential mortality or morbidity of the misdiagnosed patient. Because the sputum samples were from patients that were suspected of TB, SSM is showing 100% specificity. It has been shown that SSM has high specificity in areas with high incidence of TB.

### 4.4. SSM vs. NCBA

Out of the 500 samples, 80 samples were positive in both NCBA and Xpert MTB/RIF, generating a sensitivity of 100% and specificity of 100% for NCBA with respect to the Xpert system as gold standard. Samples that were positive in Xpert were the same samples that were positive in NCBA, giving 100% concordance with the reference standard. Based on Table 3, NCBA can detect very low bacterial load that is not detectable by SSM. SSM is only able to detect 3 out of 22 at low level, 14 out of 29 at medium level, and 15 out of 19 at high level, while NCBA is able to detect all samples at all levels, matching the detection performance of the Xpert system. This increased sensitivity is hypothesized to be due to the concentration effect by the GMNPs.

### 4.5. NCBA vs. Xpert MTB/RIF

Results show that NCBA has 100% concordance with Xpert MTB/RIF, thus making NCBA a potential alternative to the Xpert system. Results from the Xpert system in Table 3 show a wide range of detection: very low, low, medium, and high bacilli concentrations. Blakemore et al. reported that the Xpert system’s lowest detection limit (with 95% confidence) was 131 CFU/mL with a dynamic range of 10^2^ to 10^7^ CFU/mL [26]. Given the 100% agreement, NCBA therefore has the potential of detecting AFB in the same dynamic range and limit of detection as Blakemore’s report. Specifically, the average bacterial load of this cohort of samples could be in the range of 10^2^ to 10^6^ CFU/ mL, which implies that NCBA can detect as low as 10^2^ CFU/mL of AFB in the sputum sample. This level of detection is two orders of magnitude lower when compared to SSM, which requires at least 10^4^ CFU/mL for a reliable result.

When comparing the performance between NCBA and Xpert MTB/RIF, NCBA is accomplished in 10–20 min, while Xpert takes about 2 h. The main advantages of NCBA over the Xpert system are: (1) no power supply requirement, (2) no major instrumentation, (3) no cold storage requirement, (4) test results in 10–20 min, and (5) costs only $0.10/test.

## 5. Conclusions

This paper demonstrates the successful validation of the NCBA tests in 500 samples by the Xpert MTB/RIF, with the results showing perfect agreement. With the Xpert system as the gold standard, the sensitivity of NCBA is in accordance with the Xpert system (100%), while SSM’s sensitivity is only 40%. NCBA could detect very low AFB concentration at 10^2^ CFU/mL, two orders of magnitude lower than SSM. This nanotechnology-based detection is rapid (10−20 min), inexpensive ($0.10/test), simple, and easily scalable. According to Nepal’s Ministry of Health, a TB diagnostic test with 70% sensitivity (and treatment cure of 85%) would save 300,000 lives over the next five years [3]. This NCBA technique has a high potential to support and transform the TB control program in Nepal and in other high-prevalence low-resource countries. Implementation in rural areas would help to increase case finding and case notification, and would support programs targeted against drug-resistant TB. Nepal has close to 600 microscopy centers that can support the immediate implementation of this technology in the country. Similarly, it is applicable in many of the high TB-burden countries. This technique can be performed in rural communities and at the first point-of-contact by patients in the healthcare system. Results are obtained in less than 30 min, allowing for a quick turnaround time for treatment in a single clinical encounter. Globally, Desikan hypothesized that a universally accessible and rapid detection method with a sensitivity of 85% and specificity of 97% could potentially save 392,000 adjusted lives annually [9]. This NCBA technology is poised to enhance the “End TB Strategy” towards a TB-free world.

## 6. Ethics Approval

This study was approved by the Institutional Review Board of Kathmandu University Dhulikhel Hospital, Kathmandu, Nepal. The sputum samples used were left-over from patients who went to the hospital and who were suspected of having TB. Nevertheless, patients in the study have provided written informed consent.

## Figures and Tables

**Table 1:** Results using Xpert MTB/RIF as the gold standard for true tuberculosis (TB) cases and non-TB cases.

SSM Test	True TB Cases	Non-TB Cases	NCBA Test	True TB Cases	Non-TB Cases
Positive test	32	0	Positive test	80	0
Negative test	48	420	Negative test	0	420

**Table 2:** Comparison of diagnostic performance.

Technique	Xpert MTB/RIF as the Gold Standard, % (95% CI)				
	Sensitivity	Specificity	PPV	NPV	Accuracy
**SSM Test**	40 (29–52)	100 (99–100)	100	90 (88–91)	90 (87–93)
**NCBA Test**	100 (95–100)	100 (99–100)	100	100	100 (99–100)

**Table 3:** Detection limit and dynamic range of detection of the two techniques with respect to the Xpert MTB/RIF categories.

Xpert MTB/RIF Categories	Very Low	Low	Medium	High	Total
Xpert MTB/RIF	10	22	29	19	80
NCBA	10	22	29	19	80
SSM	0	3	14	15	32
% Detection (NCBA/Xpert)	100%	100%	100%	100%	
% Detection (SSM/Xpert)	0%	14%	48%	79%	
